# Bacterial Spot of Tomato and Pepper in Africa: Diversity, Emergence of T5 Race, and Management

**DOI:** 10.3389/fmicb.2022.835647

**Published:** 2022-04-18

**Authors:** Mustafa Ojonuba Jibrin, Sujan Timilsina, Gerald V. Minsavage, Garry E. Vallad, Pamela D. Roberts, Erica M. Goss, Jeffrey B. Jones

**Affiliations:** ^1^Tree Fruit Research and Extension Center, Washington State University, Wenatchee, WA, United States; ^2^Department of Crop Protection, Ahmadu Bello University, Zaria, Nigeria; ^3^Plant Pathology Department, University of Florida, Gainesville, FL, United States; ^4^Gulf Coast Research and Education Center, University of Florida, Wimauma, FL, United States; ^5^UF/IFAS Southwest Florida Research and Education Center, Immokalee, FL, United States; ^6^Emerging Pathogens Institute, University of Florida, Gainesville, FL, United States

**Keywords:** bacterial spot, tomato, pepper (*Capsicum annum* L.), Africa, *Xanthomonas*, Management

## Abstract

Bacterial spot disease was first reported from South Africa by Ethel M. Doidge in 1920. In the ensuing century after the initial discovery, the pathogen has gained global attention in plant pathology research, providing insights into host–pathogen interactions, pathogen evolution, and effector discovery, such as the first discovery of transcription activation-like effectors, among many others. Four distinct genetic groups, including *Xanthomonas euvesicatoria* (proposed name: *X. euvesicatoria* pv. *euvesicatoria*), *Xanthomonas perforans* (proposed name: *X. euvesicatoria* pv. *perforans*), *Xanthomonas gardneri* (proposed name: *Xanthomonas hortorum* pv. *gardneri*), and *Xanthomonas vesicatoria*, are known to cause bacterial spot disease. Recently, a new race of a bacterial spot pathogen, race T5, which is a product of recombination between at least two *Xanthomonas* species, was reported in Nigeria. In this review, our focus is on the progress made on the African continent, *vis-à-vis* progress made in the global bacterial spot research community to provide a body of information useful for researchers in understanding the diversity, evolutionary changes, and management of the disease in Africa.

## Introduction

Tomato (*Solanum lycopersicum* L.) and pepper (*Capsicum* species) are two of the most important vegetable crops cultivated in Africa and worldwide (FAOSTAT, 2020). Egypt and Nigeria, which, respectively, rank fifth and fifteenth globally, are the largest producers of tomatoes in Africa (FAOSTAT, 2020). FAOSTAT reports statistics on pepper production in two different categories: chilies and green pepper. Ethiopia and Ghana lead the continent in the production of dry chilies and pepper; Nigeria and Egypt lead in green pepper production (FAOSTAT, 2020). Tomato and pepper production in Africa is hampered by many constraints, including pests (insect pests, nematodes, and diseases due to fungal, bacterial, and viral pathogens), low soil fertility, low input utilization, and low market prices. One of the most important constraints in production results from attack by pathogens, some of which could lead to total crop failures, where control measures are not implemented (Jones et al., 2016).

Bacterial diseases constitute a major component of the disease challenges reducing tomato and pepper yields in all production areas (Wai et al., 2015; Jones et al., 2016). Bacterial diseases such as bacterial spot (caused by several species belonging to the genus *Xanthomonas*), bacterial speck (caused by *Pseudomonas syringae* pv. *tomato*; Young et al., 1978), bacterial canker (caused by *Clavibacter michiganensis*; Davis et al., 1984), and bacterial wilt (caused by *Ralstonia solanacearum*) are among the most devastating tomato and pepper diseases (Jones et al., 2016). When they occur, the severity or level of infection of these diseases is frequently accelerated under humid conditions and could lead to severe losses when not adequately controlled.

In this review, we focus on Africa and review efforts in understanding the bacterial spot disease in this region of the world where little information is available, but recent studies suggest important findings. The bacterial spot pathogen was first isolated from tomatoes purchased at the Pretoria market in South Africa in 1914 (Doidge, 1920). Since its first report, the disease has caused devastating yield reduction globally, with yield losses up to 45 and 50% reported in Africa and the United States, respectively (Pohronezny and Volin, 1983; Kaaya and Massomo, 2003). We trace important milestones from its first published report in 1920 to the recent report of a new race in Nigeria (Jibrin et al., 2018b). Although there are recent reviews on the bacterial spot disease and its pathogens (Potnis et al., 2015; Abrahamian et al., 2021; Osdaghi et al., 2021; Potnis, 2021), we provide an African context for research progress, especially highlighting the progress that has not been emphasized in previous reviews, to provide a useful resource for future studies and management of the disease on the African continent. We hope this information provides the platform for future research on the disease and pathogens that cause bacterial spot disease on the continent and around the world.

## Discovery of One of Africa’s Oldest Bacterial Plant Pathogens

The period 1850–1915 is generally regarded as the Golden Age of Microbiology, especially for the discovery of bacteria as pathogenic agents (Blevins and Bronze, 2010; Arguelles, 2013). This period witnessed in 1876 the discovery of the first bacterial pathogen, *Bacillus anthracis*, a causal agent of anthrax in cattle and sheep. Shortly after the discovery of anthrax, fire blight of apple and pear caused by *Erwinia amylovora* was discovered by T.J. Burrill in 1877 (Glawe, 1992). In 1882, *Mycobacterium tuberculosis* was discovered by Robert Koch. The pathogen-causing bacterial spot of tomato, initially described as “canker of tomato,” was also discovered within this golden period by Ethel M. Doidge in South Africa in 1914 (Doidge, 1920). She named the bacterium *Bacterium vesicatorium*, which was rod-shaped, aerobic, gram-negative, and contained a single polar flagellum (Doidge, 1920; Stall et al., 2009). Shortly after the publication by Doidge, the disease was identified in the United States by Gardner and Kendrick, and they named the causal organism *Bacterium exitiosum* (Gardner and Kendrick, 1921). Thus, the bacterial spot pathogen was subject to international focus very early on in discovery. Based on the reports from both studies, the strains described by Doidge were feebly amylolytic, whereas the strains described by Gardner and Kendrick were strongly amylolytic (Jones et al., 1998). Soon afterward, Gardner and Kendrick established that *B. vesicatorium* also caused bacterial spot on pepper (Gardner and Kendrick, 1923). The bacterial spot disease of pepper was also described within the same period by Sherbakoff (1918). Attempts by Higgins in 1922 and Gardner and Kendrick in 1924 to differentiate these two differently named pathogens were unsuccessful (Higgins, 1922; Gardner and Kendrick, 1924). The result was the retention of the name proposed by Doidge, *B. vesicatorium*, because of the priority rule in nomenclature (Gardner and Kendrick, 1923; Stall et al., 2009). From this point onward, the taxonomic journey of the bacterial spot pathogen took different routes.

## Important Taxonomic History of the Bacterial Spot Pathogen

*Bacterium vesicatorium* appeared to be recognized as the sole causal agent of bacterial spot of tomato and pepper for a long time. However, the nomenclature was changed several times to *Pseudomonas vesicatoria* in 1925, *Phytomonas vesicatoria* in 1930, *Xanthomonas vesicatoria* in 1939, and *Xanthomonas campestris* pv. *vesicatoria* in 1980 (Stevens, 1925; Burkholder and Li, 1941; Hayward and Waterston, 1964; Dye et al., 1980; Stall et al., 2009; Potnis et al., 2015). It was realized early in the 1990s that two distinct phenotypic and genotypic groups, named A and B, existed in *X. campestris* pv. *vesicatoria* (Vauterin et al., 1990; Stall et al., 1994). Group A strains do not hydrolyze starch or pectin, whereas group B strains do hydrolyze starch and degrade pectin (Bouzar et al., 1994c). Additionally, two genetically distinct strains identified in tomatoes from the former Yugoslavia and Florida were determined to belong to groups D and C, respectively (Šutic, 1957; Jones et al., 1995; Jones et al., 2000). In an extensive genome-based study using DNA–DNA hybridization, 16S ribosomal RNA (rRNA), and restriction fragment analyses, *X. campestris* pv. *vesicatoria* was reclassified into two species (Jones et al., 2004). These species included *X. euvesicatoria* (group A) and *X. perforans* (group C) (Jones et al., 2004). In summary, four species were designated as the causal organisms of the bacterial spot disease, including *X. euvesicatoria*, *X. vesicatoria*, *X. perforans*, and *X*. *gardneri* (Jones et al., 2004; Potnis et al., 2015). Recently, *X. euvesicatoria* and *X. perforans* were described as pathovars of the same species, and the names *X. euvesicatoria* pv. *euvesicatoria* and *X. euvesicatoria* pv. *perforans* were proposed (Constantin et al., 2016). Similarly, *X. gardneri* was recently reclassified as *Xanthomonas cynarae* pv. *gardneri* and then as *X. hortorum pv. gardneri* (Timilsina et al., 2019a; Morinière et al., 2020).

## Diagnosis of Bacterial Spot of Tomato and Pepper

**FIGURE 1:**
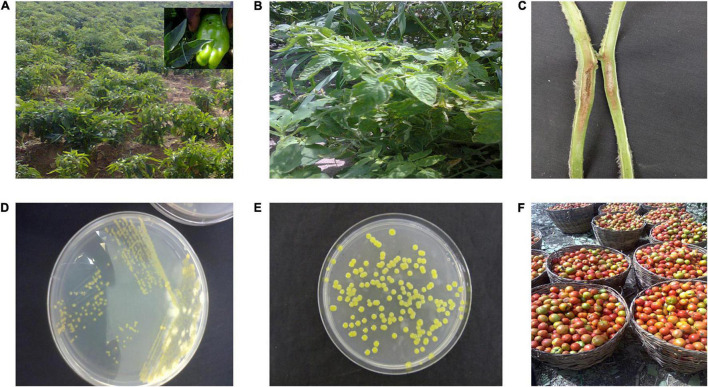
**(A)** Pepper field in northwestern Nigeria infected with bacterial spot disease. Most pepper race 6 Nigerian strains (Jibrin et al., 2014) were isolated from this field. **(B)** Tomato field in Tanzania infected with bacteria spot disease. **(C)** Pith necrosis observed in tomato stem artificially inoculated with *Xanthomonas perforans* strain Xp91-118. **(D)** Quadrant streaking to isolate pure culture of bacterial spot strains from field samples of tomato. **(E)** Pure culture obtained after sub-culturing strain from **(D)**. **(F)** Typical harvest of tomato in Nigeria. Diseased fruits are separated from healthy fruits before marketing.

Symptoms of the bacterial spot disease on tomato and pepper often occur as coalesced dark-brown necrotic lesions visible on all plant parts depending on the stage of infection (Doidge, 1920; Stall et al., 2009; Osdaghi et al., 2021; Figures 1A–C). Pith necrosis on tomatoes caused by *X. perforans* has also been described (Aiello et al., 2013; Figure 1C). On nutrient agar, the bacterium appears as yellow mucoid colonies within 24–48 h (Figures 1D,E). Pathogenicity tests often involve inoculating a bacterial suspension adjusted to 10^8^ cfu/ml from a 24-h-old culture on young plants of susceptible tomato and pepper cultivars, incubating under high humidity and at warm temperatures (28°C) for 48–72 h and observing for development of symptoms after an incubation period. Subsequently, biochemical, serological, and molecular tests have been used to help in differentiating the bacterial spot pathogens. Serological assays such as dot-immunobinding assay, immunofluorescence, and double diffusion have been used to diagnose bacterial spot of tomatoes in Egypt (Tawfik et al., 2009). However, biochemical tests are used more commonly in Africa (Shenge et al., 2007; Opara and Odibo, 2009). Such biochemical tests often include Gram-negative reaction (KOH test), positive motility test, positive xanthan gum production, hydrogen sulfide production, and unique patterns of carbon utilization patterns on BIOLOG plates (BIOLOG, Hayward, CA, United States). Sometimes, as with the first discovery, bacterial spot may be recovered from tomatoes that are designated for sale (Figure 1F shows typical packing in markets in Nigeria).

Several species-specific primers have been designed for rapid polymerase chain reaction (PCR) and quantitative PCR (qPCR) identification of bacterial spot pathogens (Koenraadt et al., 2009; Strayer et al., 2016). Although current primers are largely successful in distinguishing between the four species causing bacterial spot of tomato, it may be important to develop unique primers based on local strains in Africa because of the possibility of genetic variation. For example, the qPCR protocol based on *hrcN* primers utilized by Strayer et al. (2016) was accurate in diagnosing tomato *X. perforans* strains but not *X. euvesicatoria* strains from Nigeria (Jibrin et al., 2018b). This was because the tomato *X. euvesicatoria* strains from Nigeria had an identical *hrcN* sequence to *X. perforans*, which may have been acquired through horizontal gene transfer (Jibrin et al., 2018b). Similarly, *Xanthomonas arboricola* was identified as one of the pathogens isolated from tomatoes in Tanzania based on *gumD* and *fyuA* primers (Mbega et al., 2012a). An improved PCR technique was developed for differentiating members of pathogenic and nonpathogenic *Xanthomonas* species (Adriko et al., 2014). Such techniques could similarly be improved using available genomic resources to improve further the accuracy of diagnosing bacterial spot strains.

## Distribution of Bacterial Spot of Tomato and Pepper in Africa

**FIGURE 2:**
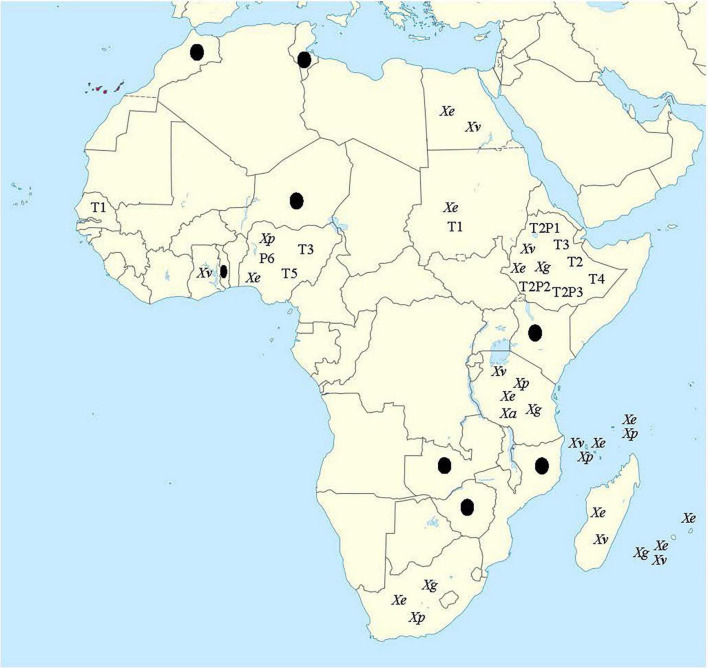
Map of Africa showing countries where bacterial spot disease has been reported on tomato and/or pepper. *Xe*, *Xp*, *Xg*, and *Xv* represent *X. euvesicatoria* (*X. euvesicatoria* pv. *euvesicatoria*), *X. perforans* (*X. euvesicatoria* pv. *perforans*), *X. hortorum* pv. *gardneri*, and *X. vesicatoria*, respectively. *Xa* represents *X. arboricola* reported only in Tanzania. Races include T1, T2, T3, T5, T2P1, T2P2, and T2P3. Countries with black dots are those for which bacterial spot have been reported, but neither species nor race was identified. Map for this figure was obtained from https://commons.wikimedia.org/wiki/File:Canary_ Islands_in_Africa_(-mini_map_-rivers).svg (date of access: 6 September 2021).

Based on routine pathogenicity and biochemical tests, the bacterial spot pathogens of tomato and pepper have been reported from a considerable number of countries in Africa and the associated Islands. Alongside South Africa, these countries include Egypt, Ethiopia, Ghana, Kenya, Malawi, Morocco, Mozambique, Niger, Nigeria, Reunion, Senegal, Seychelles, Sudan, Togo, Tunisia, Zambia, and Zimbabwe (OEPP/EPPO, 1988; Okoli and Erinle, 1989; Bouzar et al., 1994a,b; Black et al., 2001; Abd-Alla and Bashandy, 2008; Tawfik et al., 2009; Hamza et al., 2010, 2012; Opara and Odibo, 2009; Jibrin et al., 2014, 2015; Kebede et al., 2014; Lamini et al., 2015; Algam et al., 2018; Ashmawy et al., 2020). The specific species of the pathogen responsible for the disease are yet to be determined for a considerable number of countries. Figure 2 shows the map of Africa and associated Southwest Indian Ocean (SWIO) islands where bacterial spot pathogen(s) has been reported.

Identification of species of *Xanthomonas* on tomato and pepper in Africa has routinely followed the four species nomenclature. Based on this classification, all four bacterial spot *Xanthomonas* spp. have been identified in Africa. In a study of the bacterial spot pathogen in the Southwest Indian Ocean, *X. euvesicatoria*, *X. perforans*, *X. vesicatoria*, and *X. gardneri* were identified from recovered strains (Hamza et al., 2010). *X. euvesicatoria* was found in Seychelles, Mauritius, and Reunion on tomato and pepper, with three distinct groups described. Strains in the first group, which were isolated from tomatoes in Reunion and Comoros Island over a 6-year period, showed a higher population in lesions when artificially inoculated over a longer period than other strains. These strains also produced avirulent reactions on pepper cv. Aiguille. Strains in the second group were isolated from tomatoes in Seychelles and Comoros Islands and were also pathogenic on pepper cv. Aiguille. Strains in the third group were isolated from pepper in Reunion, Mauritius, Seychelles, and the Comoros Islands and were pathogenic on tomato cv. Marmande but were genetically heterogeneous based on amplified fragment length polymorphism (Hamza et al., 2010). Most strains in the third group produced a low population when inoculated on tomato cv. Marmande (Hamza et al., 2010). *X. perforans* was reported in Seychelles and Mauritius, whereas *X. vesicatoria* was found in Madagascar and Reunion. All recovered *X. perforans* and *X. vesicatoria* strains were from tomato and were pathogenic on tomato but not pepper (Hamza et al., 2010). *X. gardneri* was isolated from tomato and pepper but was found only in Reunion (Hamza et al., 2010). In a study covering tomato-growing regions of northern, central, and southern Tanzania, all four species, in addition to *X. arboricola*, were reported to cause bacterial spot of tomato (Mbega et al., 2012a). The report of *X. arboricola* causing bacterial spot of tomato in Tanzania was the first of its kind, and no other studies have identified this species as a similar bacterial spot pathogen. In Nigeria, *X. euvesicatoria* was reported in pepper in northwestern Nigeria (Jibrin, 2014; Jibrin et al., 2014). From the same region in Nigeria, an atypical *X. perforans* strain was obtained from tomato that contains alleles of genes in *X. euvesicatoria* and other unknown xanthomonads, suggesting extensive recombination (Jibrin et al., 2015; Timilsina et al., 2015). In Ghana, a survey of tomato fields in Ashanti, Brong Ahafo, and Upper East regions identified *X. vesicatoria* as the causal organism of bacterial spot disease (Lamini et al., 2015). In Sudan, *X. euvesicatoria* was reported from tomato and pepper in Blue Nile State and central Sudan (Bouzar et al., 1994a; Algam et al., 2018). *X. perforans*, *X. vesicatoria*, and *X. gardneri* are associated with bacterial spot of tomato in Ethiopia (Kebede et al., 2014). Alongside the previously described *X. euvesicatoria* by Doidge, *X. perforans* and *X. gardneri* have also been associated with bacterial spot disease in South Africa (Jibrin et al., 2018a; Jibrin, 2019).

## Epidemiology of Bacterial Spot Disease in Africa

Africa is the most tropical of all continents, and temperatures are expected to rise in the coming years if adequate measures are not taken (Engelbrecht et al., 2015; Nangombe et al., 2018). Although bacterial spot pathogens are known to cause disease globally, they often prefer warm and humid conditions for optimum disease epidemics. Early in the discovery of the disease, it was shown that the incidence of the disease was directly related to rainfall, with moist, humid conditions favorable for disease development (Doidge, 1920). Tomato and pepper cultivation in Africa is done under rain-fed and irrigation conditions and protected agriculture (Gordon, 2009; Abolmaaty and Abd El-Ghafar, 2010; NAERLS., 2012; Shenge et al., 2018). The climate of Africa coupled with the irrigation or rainfall under which tomato and pepper are cultivated inadvertently provide optimum conditions for severe disease epidemics. However, variation in epidemics due to seasons, hosts, seed source, and agronomic practices are known to occur (Abolmaaty and Abd El-Ghafar, 2010; Shenge et al., 2018). In a trial in Egypt to compare disease severity in protected agriculture (plastic house) and open field, the disease severity was significantly higher under irrigated protected agriculture than in open fields (Abolmaaty and Abd El-Ghafar, 2010). The trial also recorded that disease severity was positively correlated with average temperature and relative humidity over a 3-year period (Abolmaaty and Abd El-Ghafar, 2010). In a trial in Nigeria, the incidence and severity of the bacterial spot pathogen were more severe under rain-fed production than irrigation during the dry season (Shenge et al., 2018).

Solanaceous weeds have been reported to be hosts for the bacterial spot pathogen and may be important in disease epidemiology (Potnis et al., 2015). A recent study in Egypt identified *X. vesicatoria* associated with solanaceous plants based on pathogenicity tests and 16S rRNA analysis (Ashmawy et al., 2020). In field surveys, it was found that field sanitation influenced bacterial spot disease severity in Tanzania, with abandoned fields with no weeding having high disease severity (Shenge et al., 2010). In a recent study in Brazil, artificial inoculation of weeds with *X. euvesicatoria*, *X. perforans*, and *X. gardneri* resulted in the development of lesions typical of bacterial spot (Araújo et al., 2015; Santos et al., 2020). This is indirect evidence that the bacteria can survive on weeds. Recently, *X. perforans* strains were isolated from forest seedlings of *Eucalyptus pellita* in Indonesia (Bophela et al., 2019), suggesting that the bacteria could survive as epiphytes in forest trees.

Seeds are important in bacterial spot disease epidemiology, as they often serve as sources of inoculum for the pathogens. In Africa, the bacterial spot pathogens have been routinely isolated from seeds (Abdalla, 2000; Black et al., 2001; Opara and Odibo, 2009; Shenge et al., 2010; Mbega et al., 2012b). Isolation of pathogens from fruits is also common in Africa. Bacterial spot pathogens have been routinely isolated from fruits in Tanzania and Nigeria, with some of the strains in Nigeria being identified as *X. euvesicatoria* (Opara and Odibo, 2009; Shenge et al., 2010; Jibrin, 2014).

Wind and rain are known to disperse bacterial spot xanthomonads (McInnes et al., 1988; Bernal and Berger, 1996). However, the impact of wind-driven events such as the harmattan, which spread dry and dusty northeasterly winds from the Sahara Desert over much of West Africa into the Gulf of Guinea, have not been evaluated. Africa has also experienced recent histories of climate extremes such as cyclones, floods, droughts, and heatwaves (Reason and Keibel, 2004; Russo et al., 2016; Barbier et al., 2018). These events, which are becoming sustained in several parts of Africa, could consequently affect microbial epidemiology, including those of plant pathogens, including bacterial spot xanthomonads, as has been shown, for example, for pathogens of animals (Bannor and Ogunsan, 1988).

## Diversity in Race and Emergence of Race T5

Race determination of strains is based on the hypersensitive reaction (HR) in response to effector proteins in *Xanthomonas* strains delivered *via* the type III secretion system into host cells and recognition by specific resistance proteins in tomato or pepper (Stall et al., 2009). Table 1 shows the reactions of different bacterial spot pathogen races on the differential lines for tomato and pepper. Resistance genes in tomato include the nondominant genes *rx1*, *rx2*, and *rx3* in Hawaiian accession line H7998 and dominant *Xv3* gene in Fl216 and *Xv4* gene in *Solanum pennellii* that were introgressed into *S. lycopersicum* (Stall et al., 2009). Strains that produce HR on H7998 only are referred to as T1 strains. Only *X. euvesicatoria* strains have been identified as T1 strains to date (Stall et al., 2009; Timilsina et al., 2016). Race T2 represents strains that do not produce an HR on any of the differential lines. This includes all strains that did not contain *avrXv3*, *avrXv4*, or *avrRxv.* All *X. vesicatoria* strains and some *X. gardneri* strains belong to this race (Stall et al., 2009; Timilsina et al., 2016). Race T3 initially included strains that produced a susceptible reaction on H7998 and carried the *avrXv3* gene when expressed elicited an HR in FL216 or H7981 (Jones et al., 1988; Stall et al., 2009). Race T3 strains were determined to be *X. perforans*, as they were genetically different from *X. euvesicatoria* (Stall et al., 2009). Later, it was shown that T3 strains contain functional *xopJ4* (*avrXv4*) and elicit an HR on tomato lines with *Xv4* resistance gene (Astua-Monge et al., 2000; Stall et al., 2009). Strains that have functional *xopJ4* but nonfunctional *avrXv3* were later designated as race T4 (Timilsina et al., 2016). Pepper races are determined by the reactions of avrBs1, avrBs2, avrBs3, and avrBs4c genes in pathogens upon recognition by *Bs1*, *Bs2*, *Bs3*, and *Bs4c* resistance genes in pepper, respectively (Stall et al., 2009). The different lines used for characterization of pepper races include PI163192, PI260435, PI271322, and PI235047, which, respectively, contain the *Bs1*, *Bs2*, *Bs3*, and *Bs4* resistance genes (Stall et al., 2009; Table 1).

**TABLE 1:** Races of bacterial spot xanthomonads following Stall et al. (2009) and emergence of race T5 in Africa.

Pathogen races	Differential lines				Identification	References
Tomato						
	**Bonny Best**	**H7998**	**H7981**	**RxJop4 line**		
T1	Sus^1^	HR^2^	Sus	Sus	United States	Wang et al., 1990
T2	Sus	Sus	Sus	Sus	Brazil	Wang et al., 1990
T3	Sus	Sus	HR	HR	United States	Jones et al., 1988
T4	Sus	Sus	Sus	HR	United States	Astua-Monge et al., 2000
T5	Sus	Sus	HR	Sus	Nigeria	Jibrin et al., 2018b

**Pepper**						

	**PI163192**	**PI260435**	**PI271322**	**PI235047**		
P0	HR	HR	HR	HR		Stall et al., 2009
P1	Sus	HR	HR	HR		Stall et al., 2009
P2	HR	HR	Sus	Sus		Stall et al., 2009
P3	Sus	HR	Sus	HR		Stall et al., 2009
P4	Sus	Sus	HR	HR		Stall et al., 2009
P5	HR	Sus	Sus	Sus		Stall et al., 2009
P6	Sus	Sus	Sus	HR		Stall et al., 2009
P7	Sus	HR	HR	Sus		Stall et al., 2009
P8	Sus	HR	Sus	Sus		Stall et al., 2009
P9	Sus	Sus	HR	Sus		Stall et al., 2009
P10	Sus	Sus	Sus	Sus		Stall et al., 2009

*^1^Sus stands for susceptible reaction. ^2^HR stands for hypersensitivity reaction.*

Earlier studies of race distribution of bacterial spot of tomato and pepper strains from the Sudan and Senegal identified T1 races (Bouzar et al., 1994a,b; Jones et al., 1998). Pepper race 6 and tomato race T3 were previously identified in Nigeria (Jibrin et al., 2014, 2015). The initial race test did not include the Xv4 (also referred to as RXopJ4 differential line), and those strains were originally designated as T3 strains (Jibrin, 2014; Jibrin et al., 2015). Subsequently, it was shown that these strains, with a representative NI1 strain whose genome was sequenced, lacked *xopJ4* and would not elicit an HR on the RXopJ4 differential line (Jibrin et al., 2018b). These strains produce a new set of reactions on the available differential lines, and we propose that they should be designated as T5 race (Table 1). The highest diversity in races was reported in Ethiopia, where races T2, T2P1, T2P2, T2P3, T3, and T4 have been reported (Kebede et al., 2014). The races of bacterial spot xanthomonads that occur in other African countries are yet to be determined probably because race determination requires the use of race differential lines that are not widely available on the continent. The emergence of NI1 race T5 in northwestern Nigeria and multiple races within the central region of Ethiopia may point to unique and unsampled diversity in Africa. For example, Hamza et al. (2010) reported differential pathogenicity when *X. euvesicatoria* strains from countries in the SWIO were inoculated on pepper cultivar Aiguille as opposed to the similar pathogenicity reaction on the cultivar Marmande (Hamza et al., 2010). Future studies may yet uncover unknown races and possible novel pathogen–host interaction dynamics.

## Population Structure of African Strains Based on Multilocus Sequence Analyses

Natural environments and geographies do have an impact on bacterial evolution through various interactions that lead to mutations, gain and loss of genes, and recombination (Martinez, 2009; Pearson et al., 2009; Li et al., 2014). Understanding how bacteria evolve in different environments and their effects on population structure is important from an ecological perspective and can impact disease management (Allen and Banfield, 2005; Potnis, 2021). In several studies, multilocus sequence analyses indicated a unique population from Africa (Hamza et al., 2012; Kebede et al., 2014; Timilsina et al., 2015). In an analysis utilizing four housekeeping genes (*atpD*, *dnaK*, *efp*, and *gyrB*), strains from countries in the SWIO showed multiple sequence types (STs) in each species (Hamza et al., 2010). The type strains used in the study included *X. euvesicatoria* NCPPB2968 from the United States, *X. vesicatoria* LMG911 from New Zealand, *X. perforans* NCPPB4321 from the United States, and *X. gardneri* NCPPB881 (ATCC19865) from the former Yugoslavia. There were two (E1 and E3), three (P1, P2, and P3), one (G1), and two (V1 and V2) STs of *X. euvesicatoria*, *X. perforans*, *X. gardneri*, and *X. vesicatoria* strains, respectively, from countries in the SWIO (Hamza et al., 2010). Although most *X. euvesicatoria* strains were ST E1 (which included the type strain), ST E3 was only identified in some strains from Mauritius and Reunion (Hamza et al., 2010). ST P1 included *X. perforans* type strain and was comprised of most of the strains from Seychelles, except for a single strain that was assigned to ST P2 (Hamza et al., 2010). A clonal population was inferred for *X. gardneri*, as there was no observed diversity in the sequenced genes. Most *X. vesicatoria* strains belonged to ST V1, including the reference strain. However, strains from Madagascar and Reunion belonged to ST V2 and were identical to strains originating from Europe (Hamza et al., 2010). Interestingly too, there was evidence of recombination events in the *atpD* gene of *X. euvesicatoria* strains (Hamza et al., 2010). In a separate multilocus sequence analysis (MLSA) study based on the same housekeeping gene set, a tomato strain from Zambia (LMG927) and two tomato strains originating from the United States (ICMP110 and LMG10429) were shown to be more closely related to *X. campestris* pv. *raphani* and were pathogenic on solanaceous species and radish (Hamza et al., 2012).

Following the proposal for a standardized and uniform set of housekeeping genes for MLSAs to aid the continued study of populations of xanthomonads, subsequent studies utilized partial sequences of six genes, including *fusA*, *gapA*, *gltA*, *gyrB*, *lacF*, and *lepA* (Almeida et al., 2010). MLSAs utilizing this scheme identified a diverse population of bacterial spot pathogens consisting of *X. vesicatoria*, *X. perforans*, and *X. gardneri* in Central Ethiopia (Kebede et al., 2014). The reference strains used in the studies included *X. euvesicatoria* 85-10 from the United States, *X. perforans* 91-118 from the United States, *X. gardneri* ATCC19865 from the former Yugoslavia, and *X. vesicatoria* ATCC35937 from New Zealand. Two multilocus haplotypes were identified in *X. vesicatoria* strains from Ethiopia, whereas a third haplotype for the species was identified in Reunion and Madagascar (Kebede et al., 2014; Timilsina et al., 2015). In Ethiopia, there were no sequence differences between the *X. perforans* and *X. gardneri* strains and the reference strains of both species, suggesting that the *X. perforans* strains were similar to *X. perforans* strains from the United States. There was limited variation in *X. gardneri* in general (Kebede et al., 2014). Although the diversity of the bacterial spot pathogen recovered from samples within a short distance in Central Ethiopia was interesting, as three species were identified, there was no observed recombination in multilocus sequences of the analyzed strains (Kebede et al., 2014). Studies based on partial sequences of six housekeeping genes showed that bacterial spot of tomato strains from Nigeria gave unique results (Jibrin et al., 2015; Timilsina et al., 2015). The pepper strains were easily assigned to *X. euvesicatoria* based on MLSA (Jibrin et al., 2014); however, although the tomato strains were similar to *X. perforans* based on the routinely used *hrcN* gene sequence, they were phylogenetically difficult to assign to *X. euvesicatoria* or *X. perforans* based on MLSA (Jibrin et al., 2015; Timilsina et al., 2015). Specifically, an unusual recombination event was identified in the sequenced genes of the tomato strains from Nigeria. The partial sequences of *gltA* and *lacF* genes were identical to *X. euvesicatoria*, whereas the *fusA* and *gyrB* genes were identical to *X. perforans*. However, *gapA* and, more significantly, *lepA* genes were unique. The *lepA* gene was not related to any bacterial spot xanthomonad *lepA* gene (Timilsina et al., 2015). MLSAs failed to resolve the taxonomy of the tomato strains from Nigeria, and they were termed “atypical *X. euvesicatoria*” strains (Jibrin et al., 2015; Timilsina et al., 2015).

Population structure of African strains based on these three studies in the SWIO, Ethiopia, and Nigeria suggests unique events that drive the type and population of bacterial spot pathogens in each country. In all three studies, sampling was done within a limited area of the countries surveyed, and there is an opportunity for more studies. These types of studies utilizing multilocus analyses may be an important front for studying bacterial spot populations in Africa.

## Genomic Insights of African Strains of Bacterial Spot Xanthomonads

The reference genomes of the four species causing bacterial spot disease have been sequenced, and none of the reference strains were isolated in Africa (Thieme et al., 2005; Potnis et al., 2011). Analyses of the sequenced genomes provided important insights into types of secretion systems, effectors, lipopolysaccharide (LPS) clusters, copper resistance genes, and species-specific genes that underpin pathogen evolution and host–pathogen interaction (Thieme et al., 2005; Potnis et al., 2011).

**FIGURE 3:**
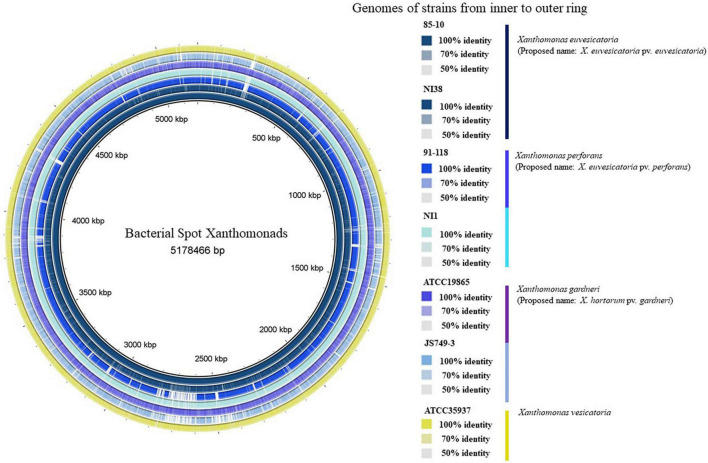
Comparisons of reference strains of each bacterial spot species against sequenced representative strains from Africa. From innermost to outermost ring: *Xanthomonas euvesicatoria* 85-10, *X. euvesicatoria* NI38, *X. perforans* 91-118, *X. perforans* NI1, *X. gardneri* ATCC19865, *X. gardneri* JS749-3, and *X. vesicatoria* ATCC35937. There was no representative African *X. vesicatoria* strain, as no known strain has been sequenced. Varying color gradients in percentage identities are based on BLAST match of a minimum of 50% percentage identity (1e-5) of genomic regions against genome of strain 85-10 as shown in key. Because BLAST matches are calculated using 85-10 as reference sequences, regions that are absent from genome of strain 85-10 but present in one or more of other genomes are not displayed. Vertical lines in key are, respectively, colored according to color of each genome in ring. Circular genome was produced using BLAST Ring Image Generator (BRIG; Alikhan et al., 2011).

Genomes of *X. euvesicatoria*, *X. perforans*, and *X. gardneri* from Africa have been sequenced (Richard et al., 2017a; Jibrin et al., 2018a,b; Jibrin, 2019). Figure 3 shows the comparison of representative African strains with the reference strains of the bacterial spot xanthomonads. The first complete genomes of bacterial spot strains from Africa were those of *X. euvesicatoria* strain LH3 and *X. gardneri* strain JS749-3 from Mauritius and Reunion, respectively (Richard et al., 2017a). The genome of strain NI1 from Nigeria was the first *X. perforans* genome from Africa that was sequenced and, alongside the genome of *X. euvesicatoria* strain NI38, were also the first genomes of African bacterial spot xanthomonads that were described in detail (Jibrin et al., 2018b). Recombination based on whole-genome sequencing among the bacterial spot pathogens was initially described in the genome of the Nigerian *X. perforans* strain, NI1 (Jibrin et al., 2018b). Subsequently, recombination was also shown in the genomes of *X. perforans* strains from Florida and Alabama in the United States (Newberry et al., 2019; Timilsina et al., 2019b). Notably, the core genome phylogeny and pangenome matrix suggest that the NI1 strain was more like *Xanthomonas axonopodis* pv. *allii* (now *X. euvesicatoria* pv. *allii*) and was intermediate between previously described *X. euvesicatoria* and *X. perforans* strains (Jibrin et al., 2018b). It was, therefore, initially thought that the Nigerian tomato strains may have been a result of the host jump between onion (*Allium* species) and tomato. The genome of strain JS749-3 from Reunion in 1997 was the first sequenced *X. gardneri* strain that was described to have the copper resistance *copLAB* gene cluster (Richard et al., 2017a). Similarly, the plasmids of both LH3 and JS749-3 contain the *cusAB-smmD*, *czcABCD*, and *arsBHCR* resistance gene clusters, except that LH3 lacks the latter gene cluster (Richard et al., 2017a). Interestingly, genome mining showed that the spread of these mobile element-associated copper resistance genes and the plasmid carrying them is restricted among bacteria within the *Xanthomonas* genus (Richard et al., 2017b). These gene clusters are important in the survival of the bacteria under heavy metal conditions (Richard et al., 2017a,b). We further provide a detailed description of variation in the secretion systems, LPS cluster, and effectors of African strains compared with the reference strains for each species.

## Secretion Systems

**FIGURE 4:**
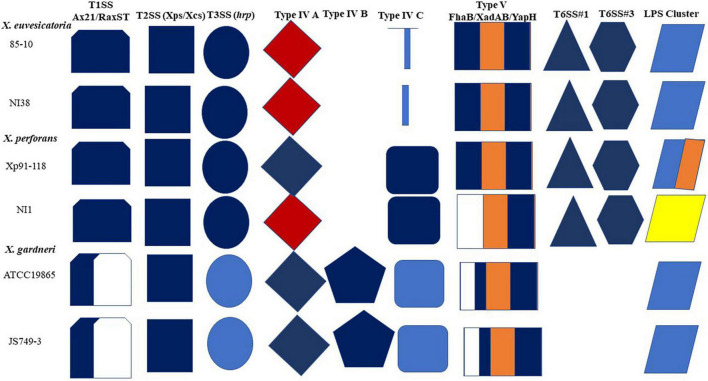
A diagrammatic representation of secretion systems and LPS clusters in reference strains compared with sequenced African strains. Shapes and color codes follow methodology of Potnis et al. (2011) with modifications. For T1SS, dark blue color in shape shows presence of homologs of Ax21 and RaxST; white space indicates absence of RaxST. T2SS systems show presence of both Xps and Xcs systems in reference strains and African strains. T3SS shows similarity in *hrp* cluster. Unique *X. gardneri hrp* cluster is in faint blue. Type IVA shows Dot/Icm cluster in red and Vir system in deep blue. Unique type IVB cluster in *X. gardneri* is also shown, whereas type IVC is shown. T5SS shows that NI1 lacks homologs of genes encoding FhaB protein, whereas one copy is present in *X. gardneri. X. gardneri* lacks T6SS. LPS cluster of NI1 is most unique and is similar to that of *X. translucence* pv. *translucence* (Jibrin et al., 2018b).

At least six (6) secretion systems were described in the sequenced reference strains of the bacterial spot xanthomonads with varying organizations (Potnis et al., 2011). These include type 1 secretion system (T1SS), type 2 secretion system (T2SS), type 3 secretion system (T3SS), type 4 secretion system (T4SS), type 5 secretion system (T5SS), and type 6 secretion system (T6SS). We review the organization of these secretion systems in NI38, NI1, and JS749-3 compared with their reference strains, which are, respectively, *X. euvesicatoria* 85-10, *X. perforans* Xp91-118, and *X. gardneri* ATCC19865. A schematic diagram showing the organization of the six secretion systems and LPS cluster is shown in Figure 4.

Type 1 secretion system: An *ax21* (activator of XA21-mediated immunity) gene was reported to be conserved in all bacterial spot xanthomonads (Potnis et al., 2011). The *ax21* gene was predicted to encode a type 1 secreted protein that is involved in quorum sensing signaling (Lee et al., 2008). Homologs of the *ax21* gene are also found in NI38, NI1, and JS749-3 and are like the copies found in their reference strains. However, there is variability in the *raxST* gene, which encodes the putative sulfotransferase *raxST* gene necessary for recognition of XA21, which is consistent with previous findings (da Silva et al., 2004; Potnis et al., 2011). Although a different homolog of the *raxST* gene is found in both NI38 and NI1 compared with the reference strains of *X. euvesicatoria* and *X. perforans*, respectively, no *raxST* gene was found in JS749-3, which is consistent with the absence of the gene in reference *X. gardneri* genome.

Type 2 secretion system: Two T2SS clusters, the Xps and Xcs systems, are found in the reference strains of the bacterial spot xanthomonads (Potnis et al., 2011). The Xps system is activated by HrpG and HrpX and represses xylanases and proteases (Szczesny et al., 2010). However, the function of the Xcs system in bacterial virulence is unclear (Szczesny et al., 2010). Both the Xps and Xcs systems are present in all the sequenced African strains, including the reference strains.

Type 3 secretion system: A *hrp* (hypersensitivity, response, and pathogenicity) gene cluster of the T3SS is found in all sequenced reference strains (Potnis et al., 2011). The *hrp* cluster of *X. euvesicatoria* is almost identical to that of *X. perforans*, with differences in the organization of the *hpaG* and *hpaF*, which are fused in *X. perforans* but occur as separate genes in *X. euvesicatoria* (Potnis et al., 2011). Previous analyses of the T3SS gene clusters in NI1 and NI38 showed that genes in the *hrp* and *hrc* (*hrp* conserved) are similar to their reference strains, with evidence of recombination in some genes (Jibrin et al., 2018b). It was also shown that although the core and pangenome of NI1 were similar to *X. axonopodis* pv. allii, the T3SS genes of NI1 were more like *X. perforans* (Jibrin et al., 2018b). The *hrcN* gene of NI38 was notably similar to the *hrcN* gene of *X. perforans* strain 4P1S2 isolated from Italy (Jibrin et al., 2018b). The organization of the *hrp* cluster in strain JS749-3 is similar to the *X. gardneri* reference strain.

Type 4 secretion system: Two T4SS clusters, Vir and Dot/Icm types, are found in bacterial spot xanthomonads. All reference strains contained more than one copy of the T4SS cluster, but only 85-10 contain both the Vir and Dot/Icm clusters, which are found on the plasmid (Thieme et al., 2005; Potnis et al., 2011). The reference strains of *X. perforans*, *X. vesicatoria*, and *X. gardneri* lack the Dot/Icm type. Additionally, two clusters of T4SS are found in Xp91-118, with one on the plasmid and the second on the chromosome. In *X. gardneri*, two unique T4SSs are found on the plasmids, whereas one cluster is present on the chromosome (Potnis et al., 2011). Based on this information, the T4SSs in bacterial spot xanthomonads were classified as type IV A (which include the Dot/Icm type in *X. euvesicatoria* and other plasmid-borne clusters), type IV B (representing the unique clusters in *X. gardneri*), and type IV C (representing the chromosomal T4SS clusters) (Potnis et al., 2011). Like their reference strains, NI38 has both the Vir and Dot/Icm clusters, whereas JS749-3 has the sets of Vir clusters described for the reference *X. gardneri* strain. However, race T5 Nigerian *X. perforans* strain NI1 T4SS cluster is different from the *X. perforans* reference strain and has both the Vir and Dot/Icm clusters, such as *X. euvesicatoria* strain 85-10 (Figure 4).

Type 5 secretion system: The T5SS secreted proteins described in the reference genomes include FhaB, XadA, XadB, and YapH (Potnis et al., 2011). FhaB hemagglutinin is present in all the reference genomes of the bacterial spot pathogen, which is possibly inactivated by an internal stop codon (Potnis et al., 2011). Homologs of the two separate open reading frames for *fhaB* are present in NI38 and JS749-3 but absent in NI1. Homologs of *Xanthomonas* adhesin-like proteins XadA and XadB, which enhance bacterial colonization of the leaf surface and entry into hydathodes (Das et al., 2009), are also found in all the reference strains and the strains NI1, NI38, and JS743-9. YapH1 and YapH2, shown to be involved in virulence in *Xanthomonas oryzae* pv. *oryzae* at later stages of growth, are present in 85-10, NI38, Xp91-118, and NI1. However, only YapH2 but not YapH1 is present in JS749-3 and the *X. gardneri* reference strain ATCC19865.

Type 6 secretion system: Three different clusters of T6SS, named types 1, 2, and 3, are reported in xanthomonads and have been shown to be involved in host–pathogen interactions (Potnis et al., 2011). The reference strains of *X. euvesicatoria* and *X. perforans* both possess the types 1 and 3 T6SS, whereas *X. vesicatoria* has the type 3 T6SS only. No T6SS cluster was reported in the reference strain of *X. gardneri* (Potnis et al., 2011). Similarly, only types 1 and 3 T6SSs are found in NI1 and NI38. No T6SS was found in strain JS743-9.

## Lipopolysaccharide Cluster

The genes in the LPS biosynthetic locus in the bacterial spot xanthomonads are positioned between two highly conserved housekeeping genes, cystathionine gamma-lyase (*metB*) and electron transport flavoprotein (*etfA*; Patil et al., 2007; Potnis et al., 2011). The LPS cluster of *X. gardneri* reference strain is identical to the LPS cluster of *X. vesicatoria* reference strain and differs from that of *X. euvesicatoria* reference strain in the functions of the two glycosyl transferases found in the cluster (Molinaro et al., 2003; Thieme et al., 2005; Potnis et al., 2011). However, *X. perforans* evolved a novel LPS cluster (Potnis et al., 2011). The *X. euvesicatoria* strain NI38 was reported to have identical LPS cluster to 85-10, but NI1 has evolved a different LPS cluster not found in other sequenced bacterial spot strains (Jibrin et al., 2018b). The LPS cluster of strain NI1 was identical to the LPS cluster of *Xanthomonas translucens* pv. *translucens*, a pathogen that causes bacterial wilt and black chaff of barley (*Hordeum vulgare*) (Jibrin et al., 2018b; Figure 4). The LPS cluster of strain JS749-3 is identical to that of the *X. gardneri* reference strain.

## Effectors

A diverse array of effectors governs the interaction of the bacterial spot xanthomonads with their host, with 11 core effectors initially described as shared among all bacterial spot species (Potnis et al., 2011). These effectors include AvrBs2, XopD, XopF1, XopK, XopL, XopN, XopQ, XopR, XopX, XopZ1, and XopAD. Subsequent studies identified XopE2 as a core bacterial spot *Xanthomonas* effector (Schwartz et al., 2015). Comparison of the genomes of African strains of bacterial spot pathogens with the reference strains and other published genomes showed that the core effectors are conserved with few notable differences (Jibrin et al., 2018b; Table 2). For *X. euvesicatoria*, all the core effectors in NI38 have the same allele as the reference strain 85-10 except for XopQ, XopAD, and XopE2, which are different alleles. There is an early termination in XopQ of NI38, resulting in 42 amino acids in the protein sequence compared with 464 amino acids in the protein sequence of XopQ of strain 85-10, suggesting that it may be nonfunctional. In *X. perforans*, there is a difference in alleles of XopD, XopK, XopL, XopQ, XopZ1, and XopAD. XopE2, which was found to be conserved in Florida, United States field strains of *X. perforans* (Schwartz et al., 2015), is absent in NI1 and Xp91-118 (Jibrin et al., 2018b). In *X. gardneri*, there are differences in alleles for XopD, XopL, XopN, XopQ, XopAD, and XopE2 in strains JS749-3 and ATCC19865.

**TABLE 2:** Effector diversity in reference strains of *X. euvesicatoria*, *X. perforans*, and *X. gardneri* compared with African strains of same species.

Effectors in *Xe, Xv, Xp, Xg^1^*	*X. euvesicatoria* (NI38 vs. 85-10)	*X. perforans* (NI1 vs. Xp91-118)	*X. gardneri* (JS749-3 vs ATCC19865)
**Core effectors**			
AvrBs2	✓	✓	✓
XopD	✓	Different alleles	Different alleles
XopF1	✓	✓	✓
XopK	✓	Different alleles	✓
XopL	✓	Different alleles	Different alleles
XopN	✓	✓	Different alleles
XopQ	Early termination in NI38	Different alleles	Different alleles
XopR	✓	✓	✓
XopX	✓	✓	✓✓ (second copy with frameshift)
XopZ1	✓	Different alleles	✓
XopAD	Different alleles	Different alleles	Different alleles
XopE2^2^	Different alleles	Absent	Different alleles
**Effectors specific to *Xe***			
AvrBs1	Absent in NI38	Absent	Absent
XopC1	✓	Absent	Absent
XopJ12	✓	Absent	Present in JS749-3
XopJ3 (AvrRxv)	✓	Absent	Absent
XopO	✓	Absent	Absent
XopAA	✓	Absent	Absent
XopAI	✓	Present in NI1	
Effectors specific to Xp			
XopC2	Two copies in NI38	✓	Absent
XopJ4	Absent	Absent in NI1	Absent
XopAF (AvrXv3)	Absent	✓	Absent
XopAE2	✓	✓	Absent
PthXp1	Absent	Absent	Absent
**Effectors Specific to Xg**			
XopAO	Absent	Absent	Different alleles
XopAQ	Absent	Present in NI1	Absent in JS749-3
XopAS	Absent	Absent	✓(second copy with different allele)
class avrBs1	Absent	Absent	✓
AvrHah1	Absent	Present in NI1	Different allele
**Effectors specific to Xv**			
XopJ2	Absent	Absent	Absent
XopAG	Absent	Absent	Absent
XopAI	Absent	Absent	Absent
	X. euvesicatoria (NI38 vs 85-10)	X. perforans (NI1 vs Xp91-118)	X. gardneri (JS749-3 vs ATCC19865)
**Effectors common to all pepper pathogens Xv, Xe and Xg**			
XopG	Absent in NI38	Absent in NI1	Absent in JS749-3
**Effectors common to Xv and Xg but absent from Xp and Xe**			
XopAM	Absent	Absent	Different alleles
HrpW	Absent	Absent	Different alleles
AvrXccA1	Absent	Absent	✓
XopZ2	Absent	Absent	✓
**Effectors common to Xg and Xe but absent from Xp and Xv**			
XopB	✓	Absent	✓
**Effectors common to Xp and Xe but absent from Xg and Xv**			
XopE1	✓	Different alleles	Absent
XopF2	✓	✓	Absent
XopI	✓	Different alleles (NI1 has 85-10 allele)	Absent
XopP	Different alleles	Different alleles	Absent
XopV	Different alleles (NI38 has NI1 allele)	Different alleles	Absent
XopAK	Different alleles	Different alleles	Absent
XopAP	Different alleles	Different alleles	Absent
**Effectors present in Xv and Xp but absent from Xg and Xe**			
XopAR	Absent	Absent in NI1	Absent
Others^3^			
AvrBsT	Absent	Absent	Absent
XopJ6	Absent	Present in NI1	Absent
XopAF-like	Absent	Absent	Absent

*^1^Xe, Xv, Xp, and Xg represents Xanthomonas euvesicatoria (proposed name: X. euvesicatoria pv. euvesicatoria), X. vesicatoria, X. perforans (proposed name: X. euvesicatoria pv. perforans) and X. gardneri (proposed name: X. hortorum pv. gardneri), respectively. In this table, comparisons are made with the reference strains. Where there are changes in the effector contents of subsequently sequenced genomes of other strains of a species, these are also highlighted.*

*^2^XopAE, XopE2, and XopJ1, though absent in the reference strains of Xe, Xp, and Xg were later found to be present in other sequenced strains of their respective species (Schwartz et al., 2015).*

*^3^AvrBsT and XopJ6, absent in the reference strain of Xp, were found in other field strains of Xp. XopAF-like were found in some Xe strains (Schwartz et al., 2015).*

Among effectors that are specific to *X. euvesicatoria*, NI38 has a similar allele with 85-10 except for AvrBs1, which is not present in NI38. All *X. euvesicatoria*-specific effectors are absent in the *X. perforans* reference strain and NI1 except for XopAI, which is present in NI1. Similarly, the reference strain of *X. gardneri* lacks all the initially described *X. euvesicatoria*-specific effectors, but XopJ1 was later found in subsequently sequenced strains (Potnis et al., 2011; Schwartz et al., 2015). *X. gardneri* strain JS749-3 possesses XopJ1 but lacks all other *X. euvesicatoria*-specific effectors (Table 2). XopAE and XopC2, initially described as specific to *X. perforans* (Potnis et al., 2011), are present in NI38 (Jibrin et al., 2018b). Although XopAE is not present in 85-10, some sequenced strains of *X. euvesicatoria* have XopAE (Schwartz et al., 2015). Interestingly, NI1 lacks XopJ4, the effector that led to the discovery of race 4 in *X. perforans.* The absence of this effector in NI1 and NI-1-like strains defines the new race T5. Recently, a new AvrBs3-type transcription activation-like (TAL) effector, pthXp1, which enhances symptom development in tomatoes and delays pustule formation in citrus, was reported in *X. perforans* strains from Italy and the United States (Jibrin et al., 2019; Newberry et al., 2019). This TAL effector is absent in all the reference strains and the African strains (Table 2). None of the effectors that were originally described to be specific to *X. perforans* are present in all sequenced strains of *X. gardneri*, including strain JS749-3. All the originally described *X. gardneri*-specific effectors are absent in sequenced strains of *X. euvesicatoria*, including NI38. However, XopAQ and AvrHah1 are present in the *X. perforans* race T5 strain NI1, representing the first time these effectors were reported in *X. perforans* (Jibrin et al., 2018b). Subsequently, AvrHah1 was also reported in some *X. perforans* strains in the United States (Newberry et al., 2019). Interestingly, *X. gardneri* strain JS749-3 lacks XopAQ and has a different allele of AvrHah1 compared with the reference strain. None of the originally described *X. vesicatoria*-specific effectors were reported in the three strains representing the three species from Africa.

XopG, an effector present in the reference strains of *X. euvesicatoria*, *X. perforans*, and *X. gardneri*, is absent in NI1, NI38, and JS749-3. XopAM, HrpW, AvrXccA1, and XopZ2 were only found in JS749-3, with the first two effectors having different alleles from the reference strain ATCC19865. XopB is a common effector found in the reference strains 85-10 and ATCC19865, and similar alleles are found in NI38 and JS749-3, respectively. The effectors that are common in 85-10 and Xp91-118 but absent in the reference strains for *X. vesicatoria* and *X. gardneri* include XopE1, XopF2, XopI, XopP, XopV, XopAK, and XopAP (Potnis et al., 2011). These effectors are absent in JS749-3, but a different mix of alleles of the effectors is present in NI1 and NI38 (Table 2). XopAR, an effector present in the reference strains of *X. perforans* and *X. vesicatoria* but absent in *X. euvesicatoria* and *X. gardneri*, is absent in NI1. Effector AvrBsT was shown to be responsible for HR on pepper (Potnis et al., 2011). Although this effector was not present in *X. perforans* reference strain Xp91-118, multiple sequenced field strains were shown to possess a copy of AvrBsT. NI1 has a different allele of AvrBsT, which was subsequently characterized and renamed as XopJ6 (Iruegas-Bocardo et al., 2018; Jibrin et al., 2018b).

Evidently, strains from Africa possess genomic profiles that have some genetic differences from the reference strains and strains in other studies, which contributed novel insights to the bacterial spot pathogens. The sequencing and study of additional strains from Africa should provide additional information into the evolution and diversity of bacterial spot xanthomonads.

## Effect of Seeds and Seed Trade on the Spread of Bacterial Spot Disease in Africa

Seeds play central roles in vegetable production in Africa (Abdalla, 2000; Opara and Odibo, 2009). Much of the tomato and pepper production in Africa results from transplants on nurseries raised by smallholder farmers themselves (Perez et al., 2017). The seeds are often sourced from commercial outlets or research institutes, grown on farmer-made beds, and transplanted to small plots (Perez et al., 2017). Open-pollinated varieties developed by national research institutes are very common, although hybrid varieties are becoming popular (Ugonna et al., 2015; Schreinemachers et al., 2021). Hybrid seeds are imported in most countries (Ugonna et al., 2015; Schreinemachers et al., 2021). Seeds, therefore, play a significant role in disease spread in Africa.

Bacterial spot pathogens can be seed-borne, and pathogen contamination of seeds and seedlings is a major factor in disease spread (McGuire and Jones, 1989; Dutta et al., 2016; Ignjatov et al., 2017; Giovanardi et al., 2018; Abrahamian et al., 2019; Abrahamian et al., 2021). In Africa, bacterial spot pathogens have frequently been isolated from commercial seeds. In a survey of farmer-saved seeds in Tanzania, bacterial spot pathogens were isolated from seeds saved from different commercial cultivars (Black et al., 2001; Shenge et al., 2007; Mbega et al., 2012c). The 16S rRNA sequences of recovered strains were identical to *X. euvesicatoria* and *X. gardneri* sequences (Mbega et al., 2012b). In Egypt, bacterial spot pathogens have also been isolated from imported seeds (Abdalla, 2000; Ashmawy et al., 2020). One of the identified species from the imported seeds was designated as *X. vesicatoria* based on 16S rRNA (Ashmawy et al., 2020).

Obviously, 16S rRNA sequences are insufficient in confirming the species of strains, and sequencing of additional loci is necessary. However, these results, which are also accompanied by pathogenicity tests, are confirmatory that bacterial spot pathogens are associated with seed trade in Africa. In the studies by Hamza et al. (2010), there was a strong hypothesis for linking bacterial spot disease in the SWIO to the importation of contaminated tomato and pepper seeds because *X. vesicatoria* strains from Madagascar and Reunion clustered closely with strains from France and Italy (Hamza et al., 2010).

Commercially produced transplants are rare in Africa, and farmers produce their own seedlings (Perez et al., 2017). It is therefore difficult to assess the role of seedlings in the spread of bacterial spot disease. However, the bacterial spot pathogen has been isolated from tomato seedlings in Egypt (El-Hendawy et al., 2005). The role of seedlings may become important in the future if large-scale tomato and pepper production gains a foothold on the continent.

## Efforts to Manage Bacterial Spot Disease in Africa

Globally, bacterial spot disease continues to cause significant yield losses of tomato and pepper (Potnis et al., 2015). Generally, efforts to improve management of the disease have focused on different strategies, including deployment of host resistance, use of antibiotics such as streptomycin, copper bactericides, systemic acquired resistance inducers, nano-based formulations of promising bactericides, small molecules, and improvement with surfactants (Stall et al., 2009; Araújo et al., 2015; Paret et al., 2013; Griffin et al., 2017; Hassan and Zyton, 2017; Liu et al., 2019; Awad-Allah et al., 2021; Jibrin et al., 2021a; Qiao et al., 2021).

Extensive use of streptomycin and copper-based bactericides can lead to the development of streptomycin and copper-resistant strains as recorded in Florida, United States (Stall and Thayer, 1962; Ritchie and Dittapongpitch, 1991; Araújo et al., 2015). Antibiotic and copper-sensitive and -resistant strains have been reported from laboratory assays in Africa. Tomato and pepper xanthomonad strains from Sudan and Ethiopia were determined to be sensitive to streptomycin and copper (Bouzar et al., 1994a,b, c; Kebede et al., 2014; Kebede and Yesuf, 2013). In Tanzania, strains isolated from tomato from three agroecological zones were sensitive to gentamycin, streptomycin, and ampicillin (Shenge et al., 2007). Similarly, strains evaluated from tomato and pepper fields in northwestern Nigeria were sensitive to copper (Jibrin, 2014). However, *X. euvesicatoria* strain LH3 and *X. gardneri* strain JS749-3 from Reunion are copper-resistant and carry the copper resistance cluster of genes on its plasmid (Richard et al., 2017a). These results may point to local management strategies deployed in individual countries influencing the composition of copper and antibiotic resistance traits of strains or to strains being imported and spread *via* seed trade. Although several studies have demonstrated the recovery of bacterial pathogens from imported seeds, there are no reports on copper resistance profiles of strains isolated from seeds, and such a study might provide market-door information on the presence or absence of copper resistance in strains imported with seeds.

Biological control strategies have also been implemented for managing bacterial spot of tomatoes. Biological control aims to prevent pathogen infection of plants or their establishment on plants (O’Brien, 2017). However, many of the experiments with biological control carried out in Africa were on already infected plant parts. In Egypt, treatment of seeds, seedlings, and foliar sprays with *Rahnella aquatilis* was shown to be effective in reducing deleterious effects of bacterial spot disease (El-Hendawy et al., 2005). Similarly, treatment of seeds and seedlings with *Pseudomonas fluorescens* alone and in combination with acibenzolar-S-methyl reduced bacterial spot disease severity (Abo-Elyousr and El-Hendawy, 2008).

Plant extracts have also shown potential in managing bacterial spot on seeds and seedlings. Extracts of ginger rhizome, mustard, and a local Ethiopian beer have been shown to inactivate a *Xanthomonas* strain of bacterial spot of tomato (Kebede and Yesuf, 2013). In Tanzania, oils of eucalyptus (*Eucalyptus globules*) and rosemary (*Rosmarinus officinalis*) and niaouli (*Melaleuca viridiflora*) at 2% concentration were shown to inhibit *X. perforans* growth in *in vitro* assays and *in planta* evaluations (Mbega et al., 2012b). Similarly, extracts of several plants, including *Aloe vera*, *Coffea arabica*, and *Yucca schidigera*, were shown to inhibit seed-borne *X. perforans* (Mbega et al., 2012c).

In general, studies involving field trials for the management of bacterial spot disease are limited. Perhaps, the absence of streptomycin and copper-resistant strains in some countries in Africa provides a window for the careful deployment of antibiotics and copper-based formulations in bacterial spot disease management. Recent advances in small molecules and nano-formulations may provide additional opportunities for the management of bacterial spot disease in the future (Liu et al., 2019; Liao et al., 2021; Qiao et al., 2021; Srivastava et al., 2021).

## Conclusion and Recommendations for Future Studies

We present progress made on the African continent on the bacterial spot of tomato and pepper disease since its discovery in the early 20th Century. Although appreciable progress has been made, gaps still exist. The identity and variation of the bacterial spot pathogen are yet to be determined in many countries in Africa. In many countries where the disease has been reported, there is a paucity of information on the species and races causing the disease. As molecular diagnostics and sequencing tools become accessible on the continent, it is expected that this situation will improve. MLSAs have proved to be a great technique in the identification and subsequent population studies of recovered isolates (Hamza et al., 2012; Kebede et al., 2014; Timilsina et al., 2015; Osdaghi et al., 2018). In as much as Sanger sequencing remains accessible, this will be an important tool for studying the bacterial spot pathogen populations throughout Africa to provide insights into population structure. Such preliminary knowledge is important for species identification and laying a benchmark for subsequent whole-genome sequence studies.

Only genomes of isolates from Mauritius, Reunion, Nigeria, Ethiopia and South Africa have been sequenced (Richard et al., 2017a; Jibrin et al., 2018a,b; Jibrin, 2019). Sequencing of these genomes produced important “firsts” in understanding the bacterial spot pathogens. It is therefore expected that studies of additional genomes of the bacterial spot pathogen in Africa should provide significant insights into the evolution and biology of the pathogens. This could provide foundations for genome-informed host–pathogen and ecological interaction studies that are unique to production systems in Africa. Additional ecological studies of these pathogens are certainly needed. *In vitro* assays in studies conducted in Tanzania showed that *P. syringae* pv. *tomato* outgrows bacterial spot xanthomonads when grown together in nutrient yeast dextrose broth (Shenge et al., 2008). The importance of bacteriocins in the emergence of *X. perforans* and subsequent displacement of *X. euvesicatoria* have also been studied in Florida populations (Hert et al., 2005; Marutani-Hert et al., 2020). Studies on bacteriocin evolution among strains are lacking in Africa and could provide important insights into the evolution of the bacterial spot pathogens on the continent. Transcriptomics can improve understanding of the interaction of bacterial spot pathogens and their hosts (Strauss et al., 2012; Du et al., 2015; Shi and Panthee, 2020). Recently, known and novel metabolites from nonbacterial spot systems were reported in *X. perforans* (Jibrin et al., 2021b). Combining metabolomics and transcriptomics in studying isolates of the bacterial spot pathogens from Africa could provide additional insights into the diversity of molecules that are important in host–pathogen or other ecological interactions and their pathways and expressed genes.

Although much progress has been achieved with the management of the pathogen on pepper, managing the disease on tomatoes is still very difficult. Often, clean seeds and good cultural practices are advocated (Jones et al., 2016). However, as has been demonstrated in this review in studies in Tanzania and Egypt, imported and farmer-saved seeds are already often contaminated with pathogens (El-Hendawy et al., 2005; Shenge et al., 2010). Seed treatment with appropriate chemicals may therefore provide a frontline in disease management. The deployment of durable resistance is still to be achieved for bacterial spot on tomatoes. The *Bs2* gene from pepper was initially touted as a possible resistance gene against bacterial spot disease on tomatoes (Tai et al., 1999; Horvath et al., 2015). However, resistance broke down, and efforts at deploying other genes have not been successful (Horvath et al., 2012, 2015). Screening tomato accessions in Africa against the bacterial spot pathogens could reveal novel resistance genes against bacterial spot.

Communicating research findings to farmers is important to help them improve disease management. Although there may be other offline resources, only one extension fact sheet was found online that targeted Africa in this review (Massawe et al., 2012). Development of management resources and training of farmers would be key to improving the management of the disease in Africa.

## Author Contributions

MJ wrote the original draft. All authors read and accepted the manuscript.

## Conflict of Interest

The authors declare that the research was conducted in the absence of any commercial or financial relationships that could be construed as a potential conflict of interest.

## Publisher’s Note

All claims expressed in this article are solely those of the authors and do not necessarily represent those of their affiliated organizations, or those of the publisher, the editors and the reviewers. Any product that may be evaluated in this article, or claim that may be made by its manufacturer, is not guaranteed or endorsed by the publisher.
